# Probing the Antitumor Mechanism of *Solanum nigrum* L. Aqueous Extract against Human Breast Cancer MCF7 Cells

**DOI:** 10.3390/bioengineering6040112

**Published:** 2019-12-11

**Authors:** Binbing Ling, Shujun Xiao, Jinha Yang, Ying Wei, Meena K. Sakharkar, Jian Yang

**Affiliations:** 1 Drug Discovery and Development Research Group, College of Pharmacy and Nutrition, University of Saskatchewan, Saskatoon, SK S7N 5E5, Canada; bblmaya@gmail.com (B.L.); xiao.57578@gmail.com (S.X.); jina5510@gmail.com (J.Y.); 2 College of Pharmacy, Guizhou University of Traditional Chinese Medicine, Guiyang 550025, Guizhou Province, China; weiying1969@hotmail.com

**Keywords:** *Solanum nigrum* L., breast cancer, cytotoxicity, cell migration, glycolysis, gene-drug interaction network, gene-disease association network

## Abstract

*Solanum nigrum* L. is one of the major medicinal plants used to treat cancer. However, the functional mechanism of *S. nigrum* L. extract is still unknown in spite of numerous studies on its active components. In this study, we probed the potential anticancer mechanism of the aqueous extract of *S. nigrum* L. (AESN) towards human breast cancer cell line MCF7. At a concentration of 10 g/L, AESN caused 43% cytotoxicity, inhibited the migration, and suppressed the activities of hexokinase and pyruvate kinase by about 30% and 40%, respectively, towards the MCF7 cells. RT^2^-PCR analysis of a panel of 89 caner-related genes identified 13 upregulated and eight downregulated genes (>2-folds) in MCF7 cells upon AESN treatment. Gene ontology (GO) and functional disease ontology (FunDO) analyses show that the antitumor function of *S. nigrum* L. involves multiple genes and these genes are shared across other diseases or disorders.

## 1. Introduction

Breast cancer is the most prevalent cancer in women worldwide, with an estimated 2.1 million new cases and 630 thousands death in 2018 [1]. Regardless of recent advances in breast cancer treatments such as targeted therapy, prognosis for advanced-stage and recurrent patients remains poor, for example, the 5-year survival rate for stage IV breast cancer is only 22% (https://www.cancer.ca/en/cancer-information/cancer-type/breast/prognosis-and-survival/survival-statistics/?region=on). Thus, many breast cancer patients, not limited to advanced-stage patients, seek complementary and alternative medicine (CAM) treatments, such as herbal remedies, meditation and nutraceutical supplementation, in expectation of improving therapeutic efficacy, boosting immune system and reducing side effects of chemotherapy. Previous studies have shown that the prevalence of CAM usage in breast cancer patients is as high as 45% [2,3,4], and traditional Chinese herbal remedies are a large part of CAM (https://www.cancer.ca/en/cancer-information/diagnosis-and-treatment/complementary-therapies/traditional-chinese-herbal-remedies/?region=on).

*Solanum nigrum* L., commonly known as black nightshade, is regarded as a wild weed in North America. However, it is widely used as a medicinal plant in traditional Chinese medicine (TCM) and Indian Ayurdeva remedies. *S. nigrum* L. is traditionally used to treat inflammation and venous skin ulcer [5]. Recently, it is used to treat different types of cancer in combination with other medicinal herbs such as *Hedyotis diffusa*, *Radix Sophorae tonkinensis* and *Scutellaria barbata* [6]. Previous in vitro studies have shown that the aqueous extract of *S. nigrum* L. (AESN) not only inhibits cell growth of oral cancer, breast cancer, cervical cancer, liver cancer, endometrial cancer and colorectal cancer but also enhances the cytotoxicity of chemotherapy agents such as cisplatin, doxorubicin and docetaxel [7,8,9,10,11,12]. Various active ingredients, such as glycoalkaloids, polyphenols, polysaccharides, glycoproteins and lunasin peptides, have been isolated and characterized from *S. nigrum* L., for example, solamargine, a glycoalkaloid, is able to inhibit cell growth and migration and induce cell apoptosis/necrosis in gastric cancer, pancreatic cancer, lung cancer, melanoma, prostate cancer, liver cancer and cholangiocarcinoma [13,14,15,16,17,18,19].

Although isolation of active components from medicinal plants and characterize their respective mechanism of action (MOA) is commonly used in discovering and developing novel anticancer therapeutic agents, such as paclitaxel and docetaxel, these studies may not necessarily represent the real functional MOAs of the medicinal plants in CAM due to several challenges as described below. Firstly, there may be several different types of active components working together to contribute the MOA of a medicinal plant. Secondly, the concentrations of an active component used in the in vitro and in vivo biomedical studies may be much higher than its natural concentrations in medicinal plants, which, in turn, diminish the validity of such studies to represent the real MOAs of the medicinal plants. Finally, the inactive components may interfere with biological functions of the active components. Therefore, in order to establish a guideline for patient’s safe usage of medicinal plants, it is important to understand their real MOAs and side effects based on studies using the whole plant extracts rather than their active components. In this study, we probed the potential anticancer mechanism of AESN towards human breast cancer MCF7 cells.

## 2. Materials and Methods

### 2.1. Materials

Human mammary gland epithelial cell line MCF12A and breast cancer luminal A subtype cell line MCF7 were purchased from the American Type Culture Collection (Manassas, VA, USA). CytoTox96 Non-Radioactive Cytotoxicity Assay was purchased from Promega North America (Madison, WI, USA). Hexokinase Activity Assay Kit and JC-1-Mitochondrial Membrane Potential Assay Kit were purchased from Abcam Inc. (Toronto, ON, Canada). Pyruvate Kinase Activity Assay Kit was purchased from Sigma-Aldrich Canada (Oakville, ON, Canada). RNeasy Mini Kit and RT^2^ First Strand Kit were purchased from Qiagen Canada (Montreal, QC, Canada). RT^2^ Profiler PCR Array Human Breast Cancer was purchased from SABiosciences Corporation (Federick, MD, USA). All experiments in the current study were carried out in triplicate.

### 2.2. Preparation of Aqueous Extract of S. nigrum L. (AESN)

Purchase, protocol used to prepare aqueous extract of *S. nigrum* L. (AESN) and HPLC-MS characterization of AESN have been previously published [6]. Briefly, 1 g of chopped *S. nigrum* L. whole plant was boiled in 100 mL deionized water for 1.5 h, and subsequently the aqueous solution was allowed to cool down at room temperature (~23 °C) for at least 2 h. The supernatant was collected with the concentration termed as 10 g/L. The supernatant was further diluted by 2-fold using deionized water with the concentration termed as 5 g/L. Both the supernatant and its 2-fold dilution were used for cell treatments.

### 2.3. Cell Culture

Both human mammary gland epithelial cell line MCF12A and breast cancer cell line MCF7 were cultured in T-75 culture flasks under ATCC-recommended cell culture conditions at 37 °C under a humidified atmosphere (5% CO_2_) in a Forma™ Series II Water-Jacketed CO_2_ Incubator (ThermoFisher Scientific Inc., Waltham, MA, USA). Cell culture media were changed every 2–3 days.

### 2.4. Cytotoxicity Assay

For the cytotoxicity assay, either human mammary gland epithelial MCF12A cells or breast cancer MCF7 cells were collected from the T-75 cell culture flasks, re-suspended in the respective culture media, and plated in 96-well culture plates with each well containing about 8000 cells. For each cell line, the cells were allowed to attach and grow for 24 h (reaching ~70% confluence) before being treated with AESN (concentration: 5 g/L and 10 g/L) for another 72 h, which was the optimal treatment time predetermined from a pilot study. Cytotoxicity of AESN was measured using the CytoTox 96 Non-radioactive Cytotoxicity Assay. Cells treated with water were used as a negative control. The cytotoxicity was calculated using the following equation:

$$Cytotoxicity\;\left(\%\right)=\frac{Experimental-Control}{Maximum\;\left(Lysis\right)-Control}$$

### 2.5. Wound Healing Migration Assay

The breast cancer MCF7 cells were seeded on 24-well plates with about 1 × 10^5^ cells per well. A scrap was made through the MCF7 confluent monolayer using a sterile 200 µL pipette tip. The plates were then undertaken media exchange and incubated at 37 °C with water (negative control) or AESN (10 g/L) treatment. Migrated distance of the MCF7 cells was imaged under a microscope at treatment time of 18 h and 48 h, respectively.

### 2.6. Hexokinase Activity Assay

Human breast cancer MCF7 cells were cultured in 6-well plates (3 × 10^5^ cells/well) with or without AESN (10 g/L) for 24 h and then were harvested from the culture plates using the assay buffer included in the Hexokinase Activity Assay Kit. The supernatant was transferred into tubes after centrifugation at 15,000 rpm for 15 min. Hexokinase activity was assayed according to manufacturer’s instructions (Abcam Inc., Toronto, ON, Canada).

### 2.7. Pyruvate Kinase Assay

Human breast cancer MCF7 cells were cultured in 6-well plates (3 × 10^5^ cells/well) with or without AESN (10 g/L) for 24 h and then were harvested from the culture plates using the assay buffer included in the Pyruvate Kinase Activity Assay Kit. The supernatant was transferred into tubes after centrifugation at 15,000 rpm for 15 min. Pyruvate kinase activity was assayed according to manufacturer’s instructions (Sigma-Aldrich Canada, Oakville, ON, Canada).

### 2.8. JC-1-Mitochondrial Membrane Potential Assay

The JC-1-mitochondrial membrane potential assay was performed according to the manufacturer’s instructions (Abcam Inc., Toronto, ON, Canada). Briefly, the MCF7 cells were plated in a 96-well plate at a density of 1.5 × 10^4^ cells per well and allowed to attach overnight before being treated with or without AESN (concentration: 10 g/L) for another 24 h. The cells were then washed once with 1× dilution buffer include in the kit before 20 µM JC-1 stain in 1× dilution buffer was added to each well and incubated at 37 °C for 10 min. The MCF7 cells were washed with 1× dilution buffer again and the signals were read at Ex475 ± 20 nm/Em530 ± 15 nm and 590 ± 17.5 nm.

### 2.9. Real-Time RT-PCR

Breast cancer specific gene expression profiles were studied using the RT^2^ Profiler PCR Array Human Breast Cancer according to the manufacturer’s recommendations (SABiosciences Corporation, Federick, MD, USA). Briefly, total mRNA was isolated from the MCF7 cells with or without AESN (10 g/L) treatment using the RNeasy Mini Kit according to manufacturer’s instructions (Qiagen Canada, Montreal, QC, Canada). RNA purity and quantity were determined spectrophotometrically by measuring the OD_260_ and OD_260_/OD_280_, respectively. Up to 1 µg of total RNA was treated with DNase and cDNA was prepared using the RT^2^ First Strand Kit. For each analysis, the control or treated cDNA samples were mixed with RT^2^ qPCR SYBR Green Master Mixture and distributed across the PCR array 96-well plates, each of which contained 89 key genes commonly involved in the dysregulation of signal transduction and other normal biological processes during breast carcinogenesis and in breast cancer cell lines. After cycling with real-time PCR (ABI 7300, Applied Biosystems, Beverly, MA, USA), the obtained amplification data (fold-changes in Ct values of all included genes) were analyzed using the SABiosciences software.

### 2.10. Druggable Genome

Genes in the druggable genome category in the DGIdb database were downloaded from http://dgidb.org. This category is a compendium of 3860 druggable genes from resource of Bader Lab Genes, Caris Molecular Intelligence, Foundation One Genes, Gene Ontology (GO), Guide to Pharmacology Genes, Hopkins Groom, Msk-Impact, RussLampel and DGene.

### 2.11. Disease Gene Set

DisGeNet (Integrative Biomedical Informatics Group, Barcelona, Spain) is a platform reporting the gene-disease associations derived from expert curated databases and text-mined data. The file for curated disease-gene associations was downloaded from website http://www.disgenet.org/web/ DisGeNET/menu/downloads. Drug-gene interaction and disease-gene association for the 21 genes with mRNA expression changed more than 2-fold upon AESN treatment in the MCF7 cells were identified using the above two datasets, respectively. A network representing the drug-gene interaction or disease-gene association was generated using Cytoscape (https://cytoscape.org/). The network statistical parameters, such as the node degree, between-ness centrality and stress centrality, were calculated using Cytoscape (Cytoscape Consortium, San Diego, CA, USA).

### 2.12. Functional Disease Ontology (FunDO) Analysis

FunDO is extracted from the NCBI Gene Reference into Function (GeneRIF) database and contains gene-disease associations. Information about the function of a gene, along with its association with diseases, is given in GeneRIF. FunDO analyses were preformed online at http://django.nubic.northwestern.edu/fundo/ for the 21 genes with mRNA expression altered by more than 2-fold upon AESN treatment in the MCF7 cells.

### 2.13. Statistical Analysis

The experimental data were processed using Microsoft Excel 2013 (Microsoft Corporation, Redmond, WA, USA) and presented as mean ± standard deviation. Statistical analyses was performed by unpaired t-test using GraphPad QuickCalcs (GraphPad Software, San Diego, CA, USA). A *p*-value of less than 0.05 was considered statistically significant and represented by an asterisk (*).

## 3. Results

### 3.1. Effect on Cytotoxicity

**Figure 1:**
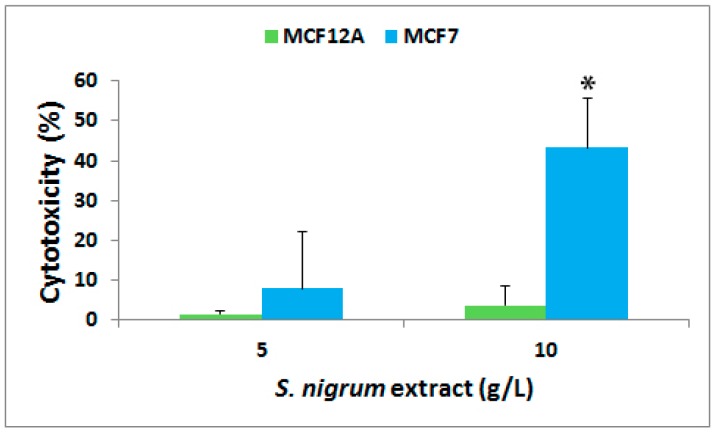
Percentage increase (mean ± standard deviation) in cytotoxicity of AESN (concentration: 5 g/L and 10 g/L) against human mammary gland epithelial cell line MCF12A and breast cancer cell line MCF7. The cytotoxicity was measured 72 h of treatment using the CytoTox 96 Non-radioactive Cytotoxicity Assay. Cells treated with water were used as a negative control.

The cytotoxicity of AESN was evaluated towards human mammary gland epithelial cell line MCF12A and breast cancer luminal A subtype cell line MCF7 at 72 h of treatment. As shown in Figure 1, AESN did not cause any cytotoxicity towards the normal breast MCF12A cells at a concentration of either 5 g/L or 10 g/L (definition of AESN concentration described in the Section 2.2). However, it imposed a cytotoxicity of 8% (*p* > 0.05) at 5 g/L and 43% (*p* < 0.05) at 10 g/L, respectively, towards the breast cancer MCF7 cells. This implicated that *S. nigrum* L. selectively targets human breast cancer cells without harming normal mammary gland cells. Furthermore, we anticipated that higher concentrated formulations of AESN would exhibit better cytotoxic effect towards the breast cancer MCF7 cells, because the AESN concentrations used in the current study are lower than the dosages normally used in patients.

### 3.2. Effect on Cell Migration

**Figure 2:**
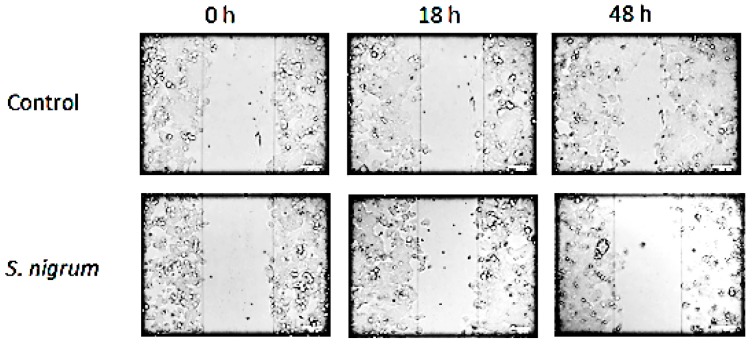
Determination of the inhibitory effect of ASEN (concentration: 10 g/L) on human breast cancer MCF7 cell migration using the wound healing migration assay.

The effect of AESN (concentration: 10 g/L) on cell migration of the breast cancer MCF7 cells was evaluated using the wound healing migration assay. As illustrated in Figure 2, MCF7 is a slow migratory cell line and AESN suppressed the migration of MCF7 cells at 48 h of treatment, which is consistent with previous studies on suppression of cancer cell migration and metastasis by *S. nigrum* L. [20,21].

### 3.3. Effect on Cell Glycolysis

**Figure 3:**
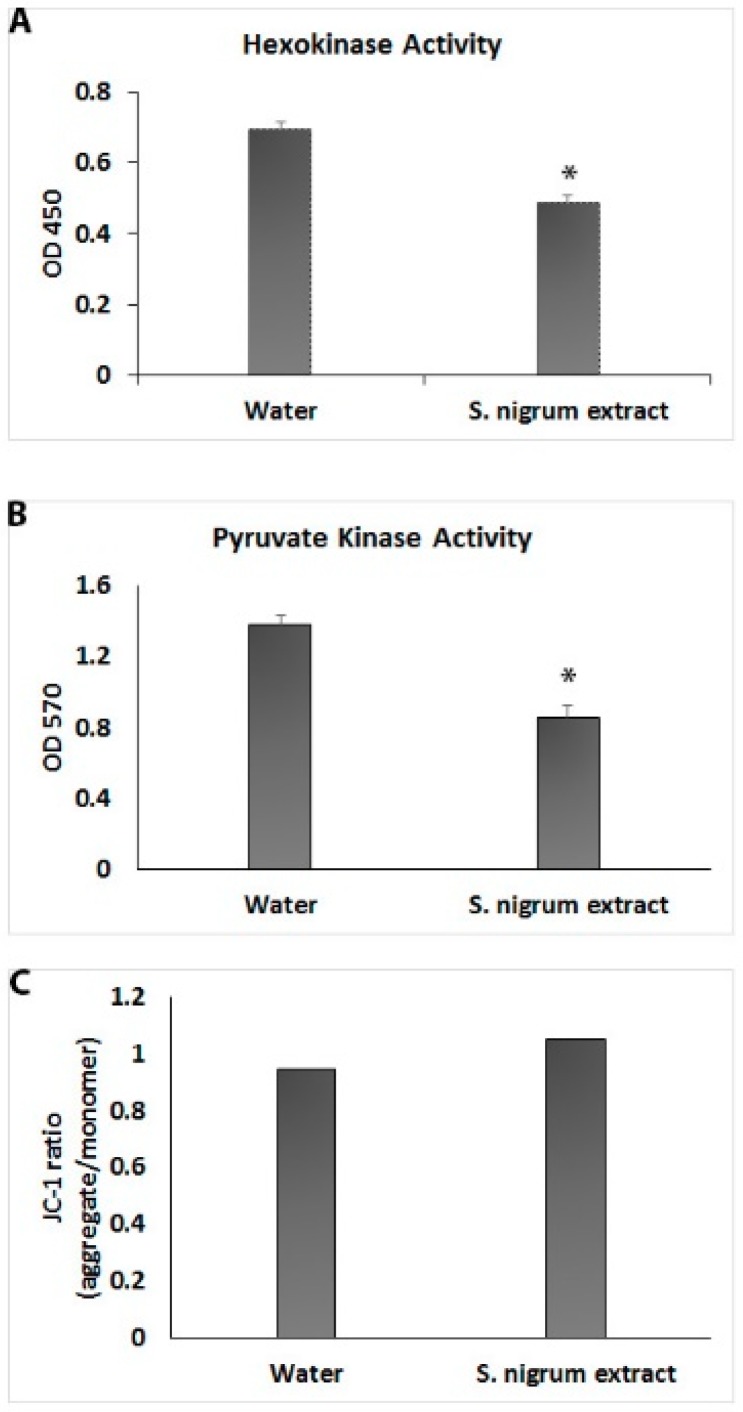
Determination of the effects of ASEN (10 g/L) on hexokinase activity (**A**), pyruvate kinase activity (**B**) and mitochondrial membrane potential (**C**) in human breast cancer MCF7 cells using the Hexokinase Activity Assay Kit, Pyruvate Kinase Activity Assay Kit and JC-1-Mitochondrial Membrane Potential Assay Kit, respectively. Treatment with water was used as a negative control.

Aerobic glycolysis (Warburg effect) is a characteristic phenomenon of cancer cells. The effect of AESN (concentration: 10 g/L) on aerobic glycolysis in the breast cancer MCF7 cells was evaluated by measuring the hexokinase activity, pyruvate kinase activity and mitochondrial membrane potential. As shown in Figure 3, the activities of hexokinase and pyruvate kinase were approximately decreased by 30% and 40%, respectively, upon AESN treatment. However, AESN did not induce any polarization or depolarization of the mitochondrial membrane in the MCF7 cells (Figure 3C). In a previous study, Lai et al. reported that AESN affected mitochondrial fission and fusion in the MCF7 cells at much higher concentrations [22]. This suggests that *S. nigrum* L. can suppress both aerobic glycolysis and mitochondrial function in the MCF7 cells under its clinical dosages.

### 3.4. Effect on the Expression of a Panel of 89-Cancer-Related Genes

In order to better understand the anticancer function of *S. nigrum* L., we investigated the effect of AESN (concentration: 10 g/L) on the expression of a panel of 89-cancer related genes using the RT^2^ Profiler PCR Array Human Breast Cancer (SABiosciences Corporation). As summarized in Table 1, genes that were upregulated by more than 2-fold are *ABCG2*, *ADAM23*, *BRCA1*, *CCNA1*, *CSF1*, *EGF*, *EGFR*, *ESR2*, *GSTP1*, *IL6*, *NOTCH1*, *SERPINE1* and *VEGFA*, and the genes that were down-regulated by more than 2-fold are *ABCB1*, *BCL2*, *CCND1*, *CDH13*, *ESR1*, *KRT19*, *PGR*, and *PTGS2*.

## 4. Discussion

The aqueous extract of *S. nigrum* L. has been shown to induce autophagy and apoptosis in cancer cells [8,9,10,11,12]. Furthermore, ethanol extract of the ripe fruits of *S. nigrum* L. induced apoptosis in human breast cancer MCF7 cells [23]. Various active ingredients of *S. nigrum* L. have been isolated and characterized for their antitumor functions. For example, an isolated glycoprotein modulated the function of transcriptional factors NF-κB and AP-1 in human breast cancer MCF7 cells [24], degalactotigonin, a steroidal glycoside, suppressed the EGFR signaling pathway in pancreatic cancer cells [25] and the Hedgehog/GLI1 signaling pathway in osteosarcoma [21], and α-solanine inhibited the epithelial-mesenchymal transition in human prostate cancer cells [26]. However, the antitumor MOA of *S. nigrum* L. aqueous or ethanol extract is still far from clear.

Consistent with previous studies, we showed that AESN possesses antitumor activity, inhibitory effect on cell migration and suppression of aerobic glycolysis towards breast cancer MCF7 cells in the current study. In addition, AESN treatment upregulated 13 genes and downregulated eight genes by more than 2-fold in the MCF7 cells. MCF7 is a luminal A subtype of breast cancer cell line, which is characterized by the presence of two hormone receptors, estrogen receptor 1 (ER1 or ERα, encoded by gene *ESR1*) and progesterone receptor (PR, encoded by gene *PGR*), and absence of human epidermal growth factor receptor 2 (HER2, encoded by gene *ERBB2*). AESN treatment downregulated the expression of gene *ESR1* by 4.3-fold and the expression of gene *PGR* by 3.5 folds. It also upregulated the expression of gene *ESR2* by 2.3-fold. Gene *ESR2* encodes estrogen receptor 2 (ER2 or ERβ), which initiates anti-proliferative effect and counteracts the function of ER1 [27]. Therefore, the current study showed that *S. nigrum* L. was able to suppress ER1- and PR-mediated cancer cell growth and might be able to achieve similar therapeutic effect as tamoxifen, an approved prodrug antagonist of ER1. Furthermore, AESN downregulated the expression of gene *ABCB1*, which encodes multidrug resistance protein 1 (MDR1), an important efflux pump in generating drug resistance in breast cancer treatment [28].

On the other hand, we observed the counteracting reactions MCF7 cells took against AESN treatment. Genes *EGF*, *EGFR* and *VEGFA*, which encode epidermal growth factor (EGF), epidermal growth factor receptor (EGFR) and vascular endothelial growth factor A (VEGF-A), were upregulated by 2.8-fold, 2.0-fold and 3.0-fold, respectively. This implies that the MCF7 cells strengthened the EGFR and VEGFR (vascular endothelial growth factor receptor) signaling pathways to promote their own growth. Concurrently, the MCF7 cells upregulated the expressions of gene *ABCG2* (encoding ATP-binding cassette super-family G member 2) by 2.5-fold and gene *GSTP1* (encoding glutathione S-transferase P) by 3.0-fold, respectively, to transport the active components of AESN out of the cells or detoxify them via glutathione conjugation within the cells. Thus, a combination of AESN and an EGFR- or VEGFR-suppressing agent might be more beneficial in killing human breast cancer MCF7 cells.

**Figure 4:**
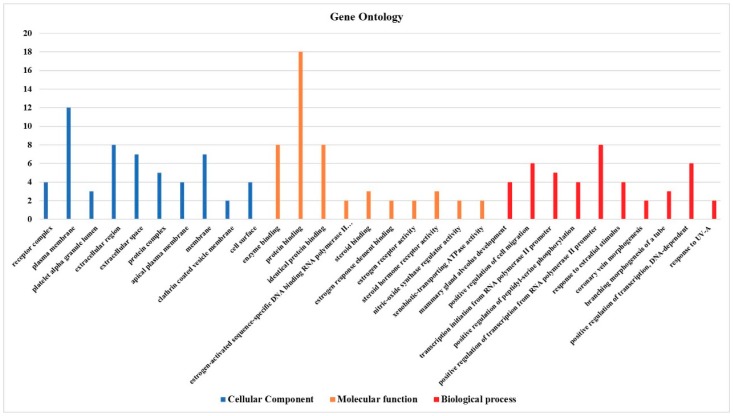
The enriched GO terms of the 21 genes, which were up- or down-regulated by more than 2 folds upon AESN (concentration: 10 g/L) treatment, in the categories of cellular component (**blue**), molecular function (**orange**) and biological process (**red**).

To get a global view of the effect of AESN on the MCF7 cells, we undertook a gene ontology (GO) study of the 21 genes, which were up- or down-regulated by more than 2-fold (Figure 4). In the category of cellular component, the top five GO terms are receptor complex, plasma membrane, platelet alpha granule lumen, extracellular region, and extracellular space. In the category of molecular function, the top five GO terms are enzyme binding, protein binding, identical protein binding, estrogen-activated sequence-specific DNA binding RNA polymerase II promoter, and steroid binding. In the category of biological process, the top five GO terms are mammary gland alveolus development, positive regulation of cell migration, transcription initiation from RNA polymerase II promoter, positive regulation of peptidyl-serine phosphorylation, and positive regulation of transcription from RNA polymerase II promoter.

**Figure 5:**
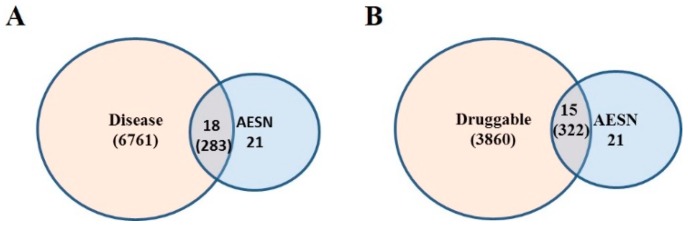
Venn diagram representations of (**A**) genes differentially expressed upon AESN treatment in MCF7 cells and disease genes and (**B**) genes differentially expressed upon AESN treatment in MCF7 cells and druggable genes.

**Figure 6:**
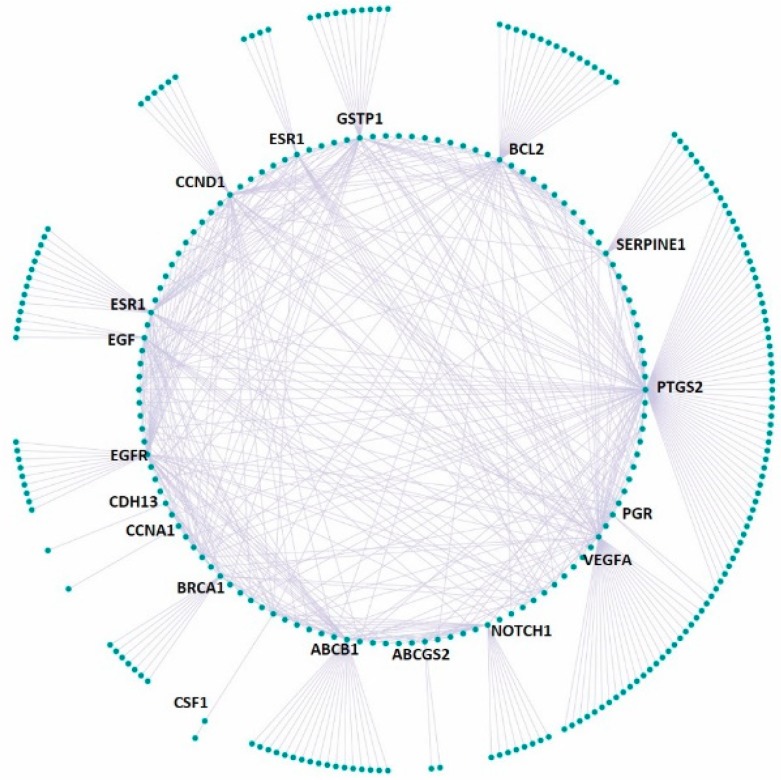
Gene-disease association network for 18 AESN-regulated genes, with each gene having at least 1 disease association and 283 unique diseases in total associated with the 18 genes.

**Figure 7:**
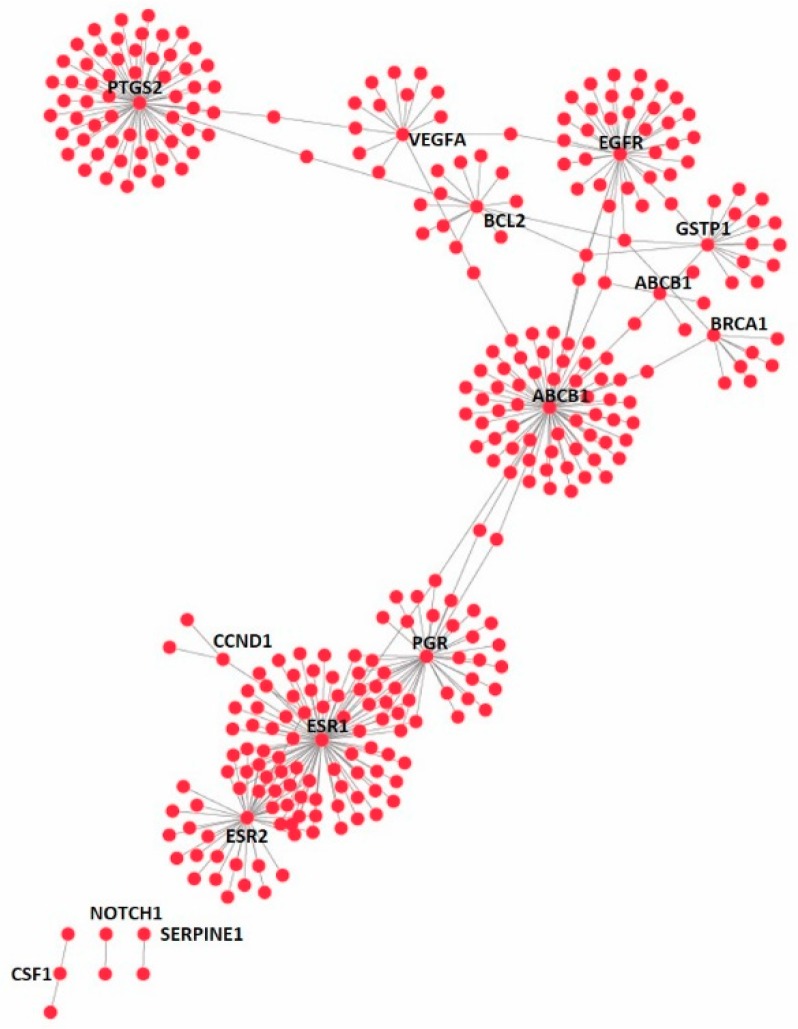
Gene-drug interaction network for 15 AESN-regulated genes, with each gene having at least 1 available drug and 322 unique available drugs in total for the 15 genes.

Furthermore, the 21 up- or down-regulated genes were searched in the curated disease gene set (DisGeNet) and the druggable genome gene set (DGIdb database), respectively, based on gene name. It was identified that 18 out of the 21 genes were found to have at least one disease association and there are 283 unique diseases in total associated with the 18 genes (Figure 5A). In addition, 15 out of 21 genes have at least one available drug and there are 322 unique available drugs in total for the 15 genes (Figure 5B). The gene-disease network for AESN is illustrated in Figure 6 and the network parameters are summarized in Supplementary Table S1. Gene *PTGS2*, which was downregulated by 2.3 folds upon AESN treatment, is involved in the maximum number (i.e., 102) of diseases. It encodes prostaglandin-endoperoxide synthase 2 (PTGS2), which is more commonly known as cyclo- oxygenase 2 (COX-2). Since PTGS2 is involved in various pathophysiological processes, including inflammation, tumorigenesis and angiogenesis [29,30,31], modulation of this target by AESN might also be beneficial in treating other diseases. The downregulation of *PTGS2* also supports the notion that *S. nigrum* L. possesses anti-inflammatory function, which has been noticed and practiced in TCM for more than one thousand years. Moreover, the gene-disease network data might even help us infer potential relationships among diseases, for example, between inflammation and breast cancer. In the gene-drug network (Figure 7), gene *ESR1* has the most available drugs (i.e., 79 drugs), followed by gene *ABCB1* with 65 available drugs and gene *PTGS2* with 59 available drugs. Interestingly, the network data show that three therapeutic drugs used to treat ER^+^ breast cancer, tamoxifen, paclitaxel and carboplatin, target four out of the 15 genes modulated by AESN. This suggests that *S. nigrum* L. could mimic the biological effects of these drugs and achieve reliable therapeutic effects towards ER^+^ breast cancer. The gene-drug network parameters are summarized in Supplementary Table S2.

**Figure 8:**
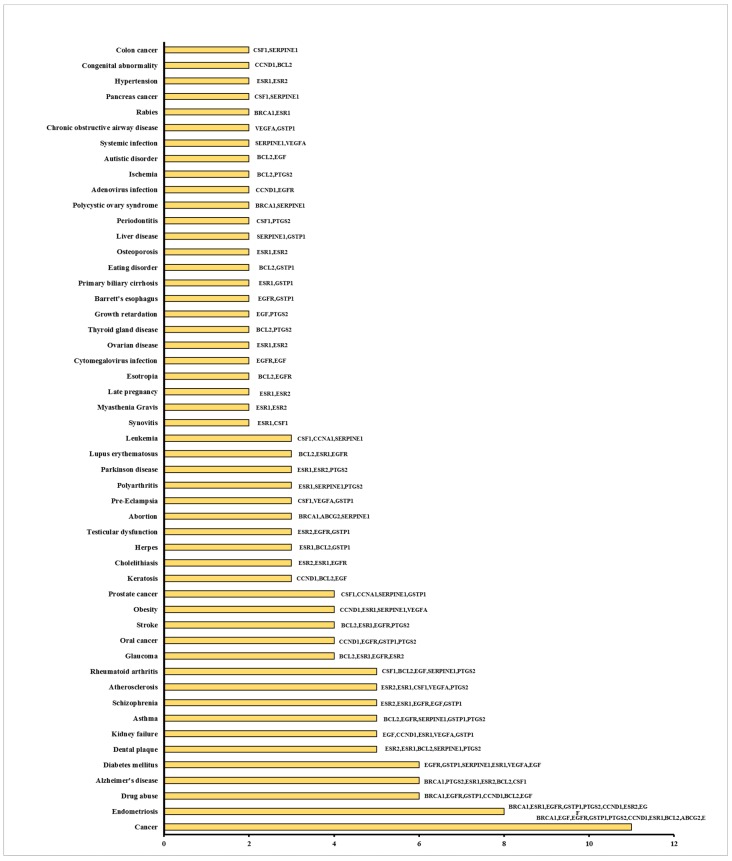
Bar chart representation of genes that are differentially expressed upon AESN treatment in breast cancer MCF7 cells and their involvement in various diseases.

We further performed Functional Disease Ontology (FunDO) analysis of the 21 genes significantly modulated by AESN. As shown in Figure 8, these genes also play important roles in other diseases/disorders, such as endometriosis, Alzheimer’s disease, diabetes and Parkinson’s disease, other than breast cancer. It is notable that genes *ESR1*, *ESR2* and *PTGS2* are shared across Alzheimer’s disease (*BRCA1*, *PTGS2*, *ESR1*, *ESR2*, *BCL2* and *CSF1*) and Parkinson’s disease (*ESR1*, *ESR2* and *PTGS2*), suggesting that they are involved in shared molecular mechanisms of neurodegeneration and inflammation in the two neurological disorders. In fact, the leaf extract of *S. nigrum* L. has shown protective effect on immobilization stress induced changes in rat’s brain [32]. Therefore, the downregulation of *ESR1* and *PTGS2* and upregulation of *ESR2* by AESN might not only provide antitumor effects against ER^+^ breast cancer but also afford protective effects towards the development and progression of Parkinson’s disease and Alzheimer’s disease. Further studies are warranted to identify whether *S. nigrum* L. would indeed alleviate the progression of these two neurological diseases. Similarly, genes *ESR1*, *SERPINE1* and *VEGFA* are shared across diabetes (*EGFR*, *GSTP1*, *SERPINE1*, *ESR1*, *VEGFA* and *EGF*) and obesity (*CCND1*, *SERPINE1*, *ESR1* and *VEGFA*), which is in line with previous reports that obesity is a risk factor of diabetes [33,34]. Nevertheless, the current data imply the potential of using *S. nigrum* L. in preventing and/or treating various diseases/disorders.

Many herbal medicines have shown beneficial effects in preventing and treating various types of cancer. For example, the aqueous extract of *Scutellaria barbata* D. Don (BZL101) has gone through phase II clinical trials and shown promising therapeutic efficacy against metastatic breast cancer [35,36]. However, cancer patients should be cautious on herbal medicine usages to avoid potential herb-drug interaction with either chemotherapy or targeted therapy agents. The current practice of warning cancer patients not to use herbal medicine during normal cancer treatment is not very effective in deterring the patients from using herbal medicine, and patients using complementary medicine were more likely to refuse additional conventional cancer therapy and experienced a higher risk of death [37,38,39]. This is partially due to patient’s fear of the disease, lack of efficacy in standard therapy, severe adverse drug reactions (ADRs) associated with the standard cancer therapy and patient’s expectation to boost the immune system. We believe that a more practical and effective strategy is to strengthen studies, accumulate sufficient scientific evidence and establish a patient guideline to ensure a safe and effective usage of traditional medicine, although such studies on whole medicinal plants are complex and complicated. As a non- or low-toxic herb, *S. nigrum* L. has been documented as one of the major anticancer medicinal plants in TCM. It is definitely worthy to undertake further studies to understand the molecular and cellular functions of this plant and explore its potential therapeutic benefits in cancer and other diseases such as Alzheimer’s disease.

Because mRNA expression does not always proportionally translate into protein expression, further studies are warranted to confirm the protein expression level of the 21 up- or down-regulated genes by AESN. In addition, we anticipate expanding our studies on AESN to other types of breast cancer cells, such as Her2-type Sk-Br-3 cells and triple-negative MDA-MB-231 cells, to fully understand the functional mechanism of AESN against human breast cancer.

## 5. Conclusions

In the current study, we demonstrated that the aqueous extract of *S. nigrum* L. (AESN) possesses cytotoxic activity, inhibitory effect on cell migration, and suppressive function on aerobic glycolysis towards human breast cancer MCF7cells. Upon AESN treatment, 13 genes were upregulated and eight genes were downregulated by more than 2-fold in the MCF7 cells. Gene ontology (GO) and functional disease ontology (FunDO) analyses show that these genes are shared across other diseases/disorders and *S. nigrum* L. might provide prevention and/or treatment benefits not only to breast cancer but also to other diseases such as Parkinson’s disease and Alzheimer’s disease.

## Figures and Tables

**Table 1:** Expression of a panel of 89-cancer related genes upon AESN treatment. This work was undertaken using the RT^2^ Profiler PCR Array Human Breast Cancer. Genes that were up- or down-regulated by more than two folds are highlighted in red and green, respectively.

Gene Name		Gene Name		Gene Name		Gene Name	
*ABCB1*	−2.5	*CST6*	−1.0	*KRT8*	−1.7	*SERPINE1*	3.1
*ABCG2*	2.5	*CTNNB1*	−1.3	*MAPK1*	1.3	*SFN*	1.4
*ADAM23*	3.9	*CTSD*	1.0	*MAPK3*	1.1	*SFRP1*	−1.0
*AKT1*	1.1	*EGF*	2.8	*MAPK8*	1.4	*SLC39A6*	−1.6
*APC*	1.3	*EGFR*	2.0	*MGMT*	−1.1	*SLIT2*	−1.3
*AR*	−1.2	*ERBB2*	−1.3	*MKI67*	1.7	*SSNAI2*	1.4
*ATM*	1.2	*ESR1*	−4.3	*MLH1*	1.1	*SRC*	1.6
*BAD*	1.3	*ESR2*	2.3	*MMP2*	−1.0	*TFF3*	−1.3
*BCL2*	−2.9	*FOXA1*	−1.1	*MMP9*	1.1	*TGFB1*	−1.1
*BIRC5*	−1.3	*GATA3*	−1.6	*MUC1*	−1.3	*THBS1*	−1.9
*BRCA1*	2.1	*GLI1*	−1.4	*MYC*	−1.0	*TP53*	1.1
*BRCA2*	1.9	*GRB7*	1.0	*NME1*	1.0	*TP73*	−1.3
*CCNA1*	3.8	*GSTP1*	3.0	*NOTCH1*	2.0	*TWIST1*	−1.4
*CCND1*	−2.3	*HIC1*	1.7	*NR3C1*	1.3	*VEGFA*	3.0
*CCND2*	1.6	*ID1*	1.3	*PGR*	−3.5	*XBP1*	−1.6
*CCNE1*	1.1	*IGF1*	−1.5	*PLAU*	1.5	*B2M*	−1.1
*CDH1*	−1.4	*IGF1R*	−1.2	*PRDM2*	1.2	*HPRT1*	1.2
*CDH13*	−2.4	*IGFBP3*	1.2	*PTEN*	1.2	*RPL13A*	−1.0
*CDK2*	−1.1	*IL6*	2.3	*PTGS2*	−2.3	*GAPDH*	1.1
*CDKN1A*	1.5	*JUN*	1.9	*PYCARD*	−1.1	*ACTB*	−1.1
*CDKN1C*	1.0	*KRT18*	−1.4	*RARB*	−1.1		
*CDKN2A*	−1.0	*KRT19*	−2.1	*RASSF1*	1.4		
*CSF1*	3.4	*KRT5*	−1.2	*RB1*	−1.0		

## Supplementary Materials

The following are available online at https://www.mdpi.com/2306-5354/6/4/112/s1, Table S1: The gene-disease network parameters, Table S2: The gene-drug network parameters. Sample Availability: Samples of the compounds are available from the authors.
