# Supplementary material for: Probing the Antitumor Mechanism of Solanum nigrum L. Aqueous Extract against Human Breast Cancer MCF7 Cells
Source: Bioengineering (Basel). 2019 Dec 11;6(4):112. doi: 10.3390/bioengineering6040112 (PMC6955812; doi:10.3390/bioengineering6040112)
Supplement: Supplementary file 1 [file bioengineering-06-00112-s001.pdf]

Supplementary materials

Table 1. The gene-disease network parameters.

Shared name	Stress	Degree	Betweenness
VEGFA	327104	61	0.21018934
SERPINE1	63058	22	0.06730401
PTGS2	612124	102	0.41865882
PGR	19374	10	0.01603795
NOTCH1	102682	20	0.06518813
GSTP1	213548	36	0.10127241
ESR2	47314	18	0.03649152
ESR1	151496	35	0.10189387
EGFR	243690	44	0.12611847
EGF	58976	19	0.03959679
CSF1	6686	2	0.00666667
CDH13	15654	8	0.00971709
CCND1	152822	34	0.08052398
CCNA1	12086	3	0.00682774
BRCA1	47820	18	0.05325804
BCL2	232832	46	0.15708805
ABCB1	156564	37	0.1348579
ABCG2	40290	8	0.01756229
**Max**	**612124**	**102**	**0.41865882**
**Average**	**139117.6**	**29.05556**	**0.091625171**

Table S2: The gene-drug network parameters.

Name	Stress	Degree	Betweenness
VEGFA	119738	14	0.18563528
SERPINE1	0	1	0
PTGS2	200482	59	0.31819843
PGR	168016	38	0.18228367
GSTP1	52190	16	0.07583039
ESR2	388122	42	0.10486904
ESR1	477798	79	0.41961072
EGFR	179772	37	0.19924206
CSF1	2	2	1
CCND1	6306	3	0.01213952
BRCA1	12958	8	0.03741261
BCL2	128632	14	0.22115822
ABCG2	7446	4	0.01818148
ABCB1	539750	65	0.66664994
NOTCH1	0	1	0
**Max**	**539750**	**79**	**1**
**Average**	**152080.8**	**25.533333**	**0.229414091**
